# Comparative Analysis of Gene Expression Level by Quantitative Real-Time PCR Has Limited Application in Objects with Different Morphology

**DOI:** 10.1371/journal.pone.0038161

**Published:** 2012-05-30

**Authors:** Natalia V. Demidenko, Aleksey A. Penin

**Affiliations:** 1 Department of Genetics, Biological faculty, Mikhail Vasilyevich (MV) Lomonosov Moscow State University, Moscow, Russia; 2 Evolutionary Genomics Laboratory, Faculty of Bioengineering and Bioinformatics, Mikhail Vasilyevich (MV) Lomonosov Moscow State University, Moscow, Russia; 3 A.A. Kharkevich Institute for Information Transmission Problems, Russian Academy of Science, Moscow, Russia; Louisiana State University Health Sciences Center, United States of America

## Abstract

qRT-PCR is a generally acknowledged method for gene expression analysis due to its precision and reproducibility. However, it is well known that the accuracy of qRT-PCR data varies greatly depending on the experimental design and data analysis. Recently, a set of guidelines has been proposed that aims to improve the reliability of qRT-PCR. However, there are additional factors that have not been taken into consideration in these guidelines that can seriously affect the data obtained using this method. In this study, we report the influence that object morphology can have on qRT-PCR data. We have used a number of *Arabidopsis thaliana* mutants with altered floral morphology as models for this study. These mutants have been well characterised (including in terms of gene expression levels and patterns) by other techniques. This allows us to compare the results from the qRT-PCR with the results inferred from other methods. We demonstrate that the comparison of gene expression levels in objects that differ greatly in their morphology can lead to erroneous results.

## Introduction

Over the past twenty years real-time qRT-PCR has become a powerful approach for the accurate quantification of gene expression. During the development of this technique from the first studies with ethidium bromide staining [1], several important improvements have been introduced. However, in spite of the increased accuracy of real-time qRT-PCR there are still several frequent errors in experimental procedures which can lead to the generation of biologically meaningless data.

In order to address this problem, a set of guidelines describing the minimum information necessary for the evaluation of qRT-PCR experiments was recently proposed [2]. These guidelines are now widely accepted in the biological science community; suffice it to say that the instructions for authors of several high-impact journals include the recommendation to follow these guidelines [e.g. 3].

Incorrect normalisation may lead to serious inaccuracy in data analysis. It is well-known that a normalisation strategy that relies on the use of reference genes (the genes for which expression is stable in all samples being compared) is preferable for real-time qRT-PCR experiments [e.g. 4, 5]. In some cases the degree of inaccuracy can reach a 10-fold error [6]. To avoid this problem, some approaches for validation were proposed, including geNorm, NormFinder, BestKeeper, qBase [7]–[10]. All of these approaches were subject to preliminary tests on human tissues, and have been applied to a wide range of other objects.

In this study we are focusing on the application of qRT-PCR to plant studies. In the case of plant studies, Brunner and coauthors [11] reported that not all of the best known reference genes are equal. Further to this, Czechowski and coauthors showed that the most frequently used reference genes are hardly appropriate for data normalisation, and proposed a number of novel reference genes [6]. To date, there are many studies in which the search and validation of reference genes are reported, but most of them are focusing on the traditionally used “housekeeping” genes, not novel candidate reference genes that have been inferred from genome-wide studies such as in [6]. This issue can be settled by obtaining ortholog sequences for novel references with the help of degenerate primers, or by searching genome/transcriptome-wide sequencing data [12] in addition to further validation. Moreover, even if reference genes have already been selected for the object, double-checking of their expression stability under experimental conditions is preferable in order to increase the accuracy of real-time qRT-PCR analysis [13].

Another group of probable source of errors is more specific, but no less dangerous, and can result in incorrect data acquisition. The qRT-PCR data generation and analysis methodology indirectly implies that the samples being compared are similar in their morphology. The extent of the applicability of qRT-PCR to comparative analysis of gene expression levels in objects which are characterised by different morphology has never been discussed. We assume that in this case the data obtained from real-time qRT-PCR could be biologically meaningless. In order to test this, conclusions based on qRT-PCR data can be compared to those based on more direct experimental evidence such as *in situ* hybridisation or gene interactions predicted by mutant analysis.

**Figure 1:**
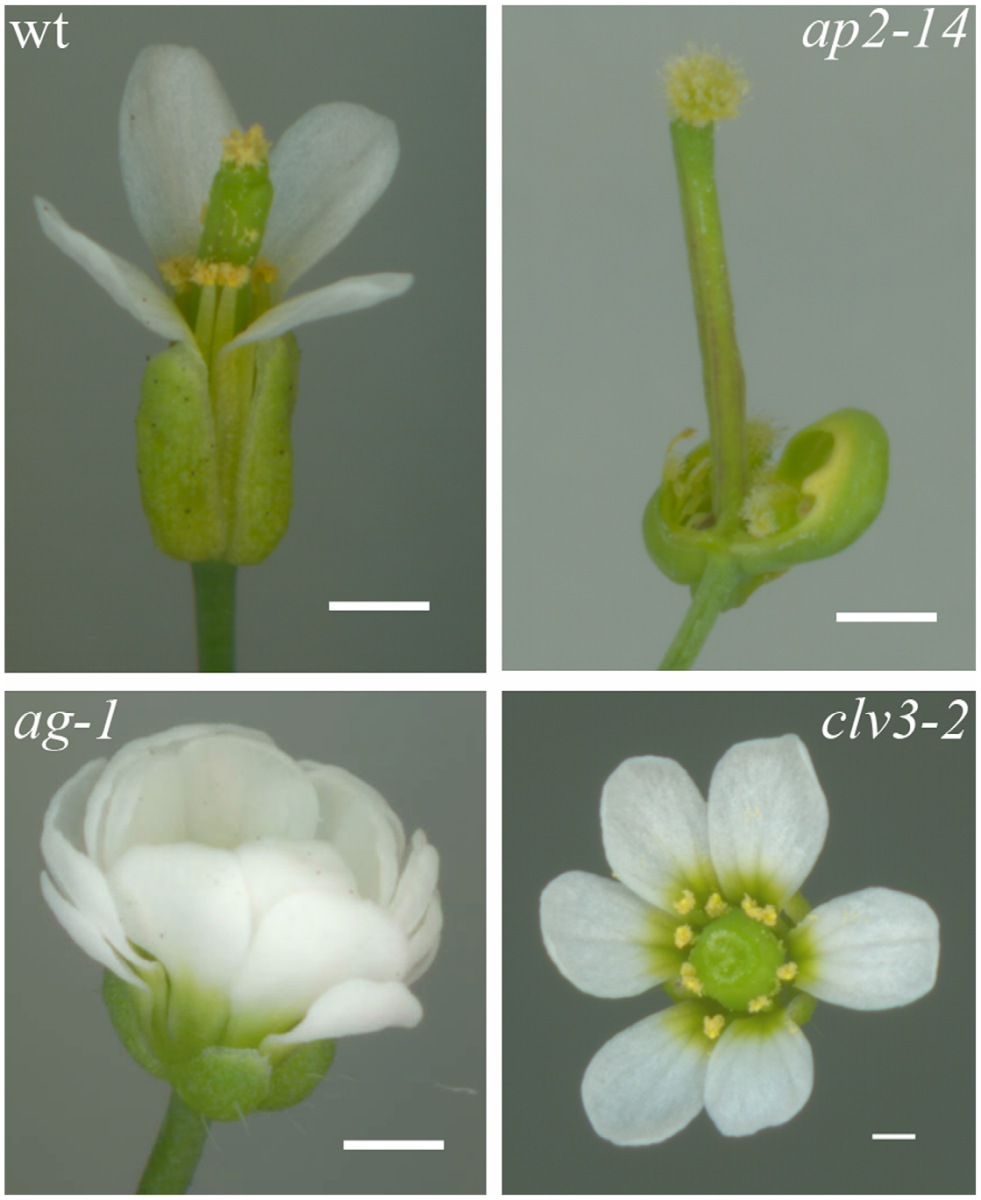
Flowers of wild type (wt) and of three single mutants of *A. thaliana*. Scale bar = 5 mm.

To investigate the influence of object morphology on the validity of qRT-PCR data we analysed the expression of genes involved in flower development and maintenance of floral meristem. Alteration of stem cell activity in the floral meristem in mutants of *Arabidopsis thaliana* is characterised by a dramatic change in floral organ number and identity (Fig. 1).

## Results

The expression levels of genes controlling floral organ identity (*AGAMOUS* (*AG*), *APETALA2* (*AP2*), *APETALA3* (*AP3*) and *PISTILLATA* (*PI*)) and regulators of meristematic activity (*WUSCHEL* (*WUS*) and *CLAVATA1, 2* (*CLV1, 2*)) were analysed in three mutants with an altered number and identity of floral organs: *ap2-14*, *ag-1* and *clv3-2*.

**Figure 2:**
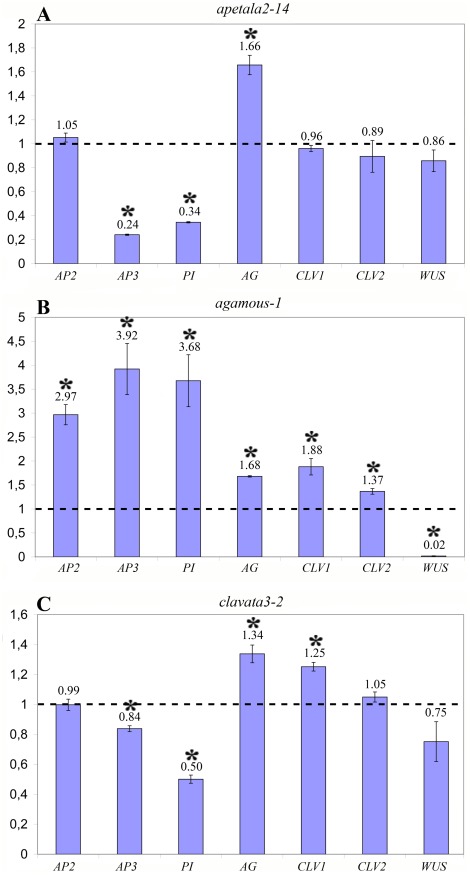
The relative expression level of flower development genes. (a) *ap2-14* mutant analysis, (b) *ag-1* mutant analysis, (c) *clv3-2* mutant analysis. Error bars represent the standard error of the mean. Asterisks indicates what values are significant to p<0.05. *AP2* - *APETALA2*, *AP3* - *APETALA3*, *PI* - *PISTILLATA*, *AG* - *AGAMOUS*, *CLV1* - *CLAVATA1*, *CLV2* - *CLAVATA2*, *WUS* – *WUCSHEL.* Dashed line indicates 1.0 expression level.

The qRT-PCR analysis of the *ap2-14* mutant revealed that expression levels of *AP3* and *PI* had decreased four-fold and three-fold respectively. The expression level of *AG* had increased by slightly more than half. The expression levels of *AP2*, *CLV1*, *CLV2* and *WUS* did not change significantly (Fig. 2A).

Gene expression levels obtained from the analysis of *ag-1* indicated that *WUS* expression was reduced by two orders of magnitude. While the expression levels of *AP3* and *PI* had increased fourfold, and *AP2* and *CLV1* levels had tripled and doubled respectively. (Fig. 2B).

In the *clv3-2* mutant no significant changes in gene expression levels were observed except for *PI,* for which the expression level was reduced two-fold (Fig. 2C).

## Discussion

Over the last twenty years, many aspects of the genetic control of development in *Arabidopsis thaliana* have been uncovered. In particular, the mechanism underlying the determination of floral organ identity (the ABC-model) and the system for the regulation of meristematic activity of the floral meristem [14], [15]. The functions and interactions of the genes involved in these processes were investigated by various methods, including the study of expression patterns by *in situ* hybridisation, the phenotypic analysis of mutants and transgenic plants and DNA-protein interactions.

The key gene responsible for the maintenance of meristematic activity is *WUSCHEL* (*WUS*) [16]. In wild type *Arabidopsis* it is characterised by a very narrow expression area. *WUS* is expressed in only a few cells in the shoot apical and floral meristem. The genes *CLV1, CLV2* and *CLV3* restrict *WUS* expression in the shoot apex [17]. In the flower meristem an additional gene, *AGAMOUS* (*AG*), acts to restrict *WUS* expression [18]. *AG* is crucial for the determination and development of reproductive organs (a C-class gene, in terms of the ABC model) [15]. *AG* expression is confined to the inner two whorls by the A-class gene *AP2* [18]; in turn, *AP2* translation in the third and fourth whorls is repressed by the microRNA *mir172* [19]. These genetic interactions create a boundary between the perianth and the reproductive organs. The *APETALA3* (*AP3*) and *PISTILLATA* (*PI*) genes express in the second and third whorls, conferring petal and stamen identity [15] and are involved in positive feedback interactions [20].

All of the mutants used in this study have already been characterised by several other methods. It is natural to expect that the changes in gene expression levels observed using qRT-PCR will be consistent with those inferred from other experiments. The main deviation from the expected was observed is shown by the *ag-1* mutant. This mutant is characterised by the lack of determination of the floral meristem, a phenotype that results from the impairment of *AG* as a negative regulator of the meristematic cell maintenance gene *WUS*. Thus an increase in the *WUS* expression level is to be expected. It has been experimentally shown that in the *ag-1* mutant, a lack of negative regulation of *WUS* results in the prolongation and slight broadening of its expression area [21]. However, the results of the qRT-PCR contradict this; instead of increased *WUS* expression, a major decrease was observed. The main explanation for such a phenomenon is the inapplicability of the basic statistical method for the calculation of relative expression data – the ddCt method [22]. This algorithm is based on the comparison between the ratio of reference genes to the expression levels of genes of interest, and indirectly implies that the expression pattern of these genes is similar in the samples being compared. However, in the case of *ag-1*, even if *WUS* expression is increased twofold the expression area of the reference genes is simultaneously increased by several orders of magnitude due to a strong increase in floral organ number. Such disproportionate results indicate that real-time qRT-PCR is incapable of providing accurate data for gene expression levels.

Another noticeable effect is the observed increase in expression levels of B-class genes (*AP3* and *PI*). This is due to the expansion of their expression region – petals and stamens – that is characteristic of the *ag-1* mutant phenotype. On the contrary, in the *ap2-14* mutant which has reduced number of floral organs the decrease in the expression of B-class genes is observed. These variations in B-class expression are directly related to their expression patterns in both cases.

The *clv3-2* mutant analysis had a similar result. *WUS* is negatively regulated by *CLV3*, thus in the case of a mutation in *CLV3* an increase of *WUS* expression is expected. However, according to qRT-PCR analysis its expression level did not change in *clv3-2* mutants. This is also associated with the mutant phenotype which is characterised by an increase in meristem activity leading to an increase in floral organ size and number. As a result, the expression area of the reference genes also increases. This change leads to the incorrect quantification of genes of interest and masks the *WUS* expression increase. This example also confirms the non-universality of the ddCt method and non-applicability of real-time qRT-PCR for such an analysis.

All of the other results obtained were consistent with the expectations based on the mutant phenotype and present data on gene function and interaction.

The errors in this approach can seriously influence the determination of final conclusions such as the identification of gene interactions or expression area. The real-time qRT-PCR method can not lead the researcher to accurately conclude whether the expression level has increased as a result of broadening its area or because it produced more mRNA. Subsequently, it is difficult to discriminate between cadastral interactions or positive/negative regulation.

In conclusion, the present study indicates that there is a problem with the application of real-time qRT-PCR. Using the common and well-studied model *Arabidopsis,* particularly mutants with altered floral morphology, we have shown the influence of this factor on the accuracy and validity of qRT-PCR results. We suggest that other cases could have similar issues (e.g. interspecific gene expression studies) and lead to incorrect conclusions. One possible way to reveal that the method is the source of error is by simultaneous gene expression analyses of various genes that are involved in mutant phenotype development. Although this cannot help to reconstruct the real data, it can indicate the errors and help to avoid gathering noisy data. Alternatively, corroboration of the real-time qRT-PCR data by other methods (e.g. RNA-seq) is also suitable for obtaining the actual data.

## Methods

### Plant Material and Biological Samples

For the gene expression analysis, *Arabidopsis thaliana* plants were grown on 1∶2 vermiculite:soil at 25°C, in 60% relative humidity under long day (16 hours light/8 hours dark) conditions. The mutant lines *clv3-2* and *ag-1* are in the Ler background thus Ler wild type plants were taken for comparison with *ag-1* and *clv3-2*. The mutant line *ap2-14* is in the Col background and Col wild type plants were used for comparison with *ap2-14*. Young inflorescences at the stage of the anthesis of the first flower were collected in two biological replicates. No specific permits were required for the described field studies.

### RNA Extraction and cDNA Synthesis

Total RNA was isolated from 50
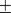
5 mg of plant material using an RNeasy Plant Kit (Qiagen, USA) with some modifications. To prevent DNA contamination, samples were treated twice with RNase-Free DNase (Qiagen, USA). The first digestion was performed according to the manufacturer’s instructions, then columns were washed with 350 µl of RW1 and the digestion was repeated. To evaluate RNA integrity, RNA was visualised on 1% SYBR-Green-stained agarose gel. Clear bands corresponding to 18 S and 28 S rRNA and the absence of a smear were observed indicating minimal degradation of RNA. The concentration of isolated RNA was calculated using a Qubit (Invitrogen, USA). The concentration of total RNA was more than 100 ng/µl among all samples. Total RNA samples were stored at −70°C with the addition of RNAse inhibitor RNasin (Sileks, Russia) and were then adjusted to the concentration of 100
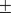
5 ng/µl for reverse transcription. First strand cDNA synthesis was performed using a “First strand cDNA synthesis kit” (Sileks, Russia) with a 24 T primer (0,4 nmol per reaction) in a 25 µl reaction mix according to the manufacturer’s protocol. Before each PCR run the cDNA samples were heated (65°C –90″, 40°C –30″) and then the cDNA products were diluted 10-fold prior to use in real-time PCR.

### qRT-PCR Conditions

Quantitative real-time PCR analysis was performed on a StepOnePlus Real-Time PCR System (Applied Biosystems, USA) using a 2.5× RT-PCR reaction mix (Syntol, Russia). Primer sequences and amplification conditions are listed in the table. To detect dsDNA synthesis EvaGreen dye was used. Each reaction was performed in a 20 µl mix containing 400 nmol of each primer and 1 µl of 1∶10 diluted cDNA. qRT-PCR conditions were five mins at 95°C, then 35 cycles of 95°C at 15 s and 62°C at 60 s. Each sample was analysed in triplicate; mean Ct values were calculated. Mean Ct dispersal for technical replicates did not exceed 0,3 cycle. To reveal the absence of contamination or primer dimers a non-template control (NTC) reaction with each primer pair was run. To ensure the absence of gDNA reverse transcription negative controls were performed with each biological sample. These no-RT control reactions were run with primers to the *CLV2* gene because these primers anneal within one exon. To obtain amplicon data a melting curve analysis was performed after each PCR run (Fig. S1). The list of analysed genes, primers and different parameters derived from qRT-PCR analysis is in Table S1.

### Gene Expression Analysis

Obtained Ct values for each sample were transformed into Cq values by the standard formula: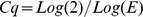, where *E* is the efficiency of the amplification of each primer pair. Amplification efficiency was calculated using Miner ver. 2.2 software [23]. The relative expression levels were calculated using the ddCt method. Relative expression levels were normalised to the geometric average of the Cq values of two reference genes: *AT4G34270* and *AT5G25760*. These genes are among the most stably expressed according to a genome-wide survey by Czechowski et al. [6].

## Supporting Information

Figure S1 **Specificity of RT-qPCR.** Melting curves generated for all genes in three technical repetitions. Low-fluorescence curves indicate NTC.
(TIF)

Table S1 **List of analysed genes, primers and different parameters derived from qRT-PCR analysis.**
(DOC)
