# Correction: Comparative Analysis of Gene Expression Level by Quantitative Real-Time PCR Has Limited Application in Objects with Different Morphology

**DOI:** 10.1371/annotation/fe0ad7f2-2921-4b13-903a-6eba19e8294b

**Published:** 2012-08-23

**Authors:** Natalia V. Demidenko, Aleksey A. Penin

There were errors in the author affiliations. The affiliations should appear as shown here:

 1 Department of Genetics, Biological faculty, M.V. Lomonosov Moscow State University, Moscow, Russia, 2 Evolutionary Genomics Laboratory, Faculty of Bioengineering and Bioinformatics, M.V. Lomonosov Moscow State University, Moscow, Russia, 3 A.A. Kharkevich Institute for Information Transmission Problems, Russian Academy of Science, Moscow, Russia 

