# Supplementary figures and images for: Comparative Analysis of Gene Expression Level by Quantitative Real-Time PCR Has Limited Application in Objects with Different Morphology
Source: PLoS One. 2012 May 30;7(5):e38161. doi: 10.1371/journal.pone.0038161 (PMC3364230; doi:10.1371/journal.pone.0038161)

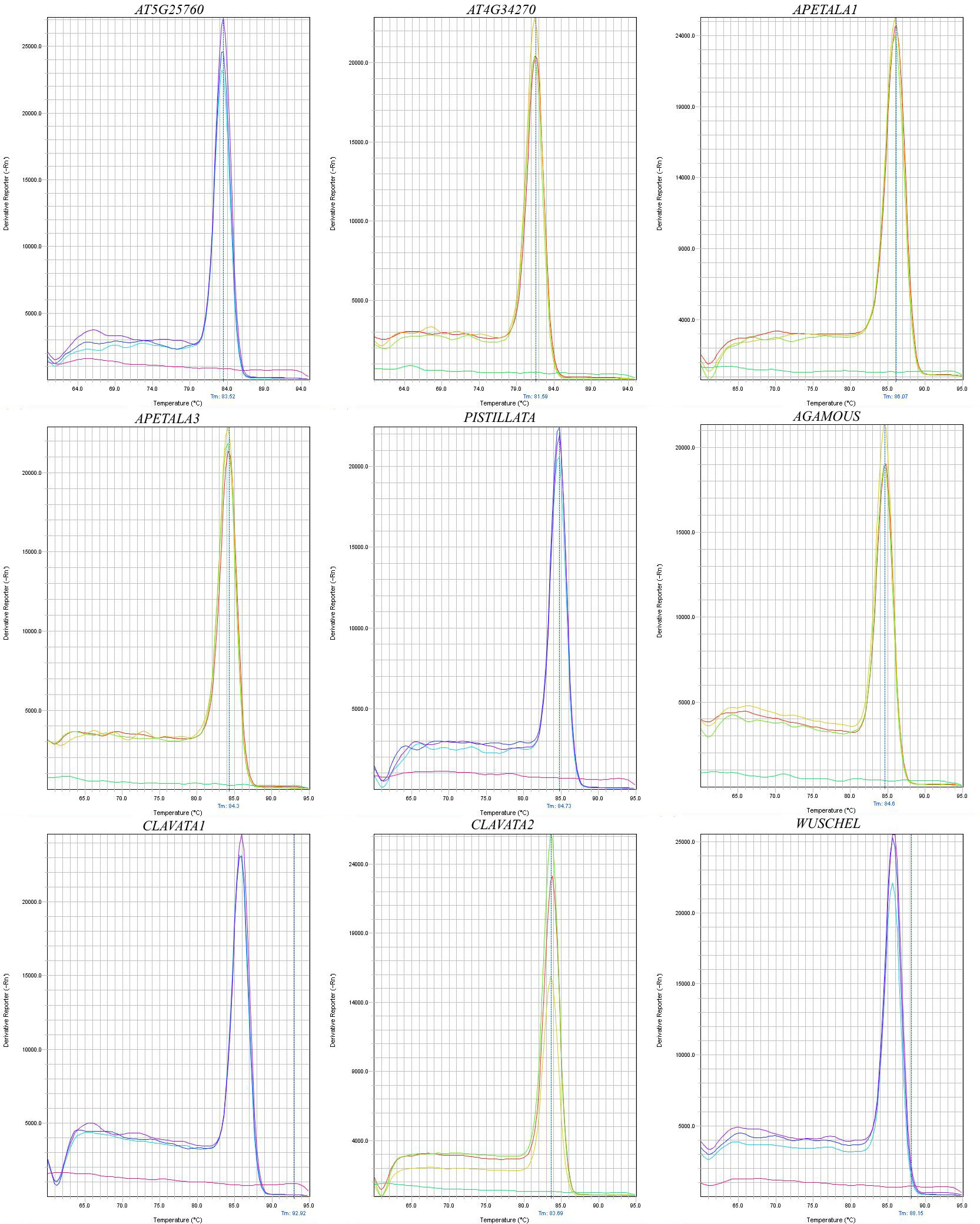

Supplement: Figure S1 — Specificity of RT-qPCR. Melting curves generated for all genes in three technical repetitions. Low-fluorescence curves indicate NTC. (TIF) [file pone.0038161.s001.tif]
