# Supplementary material for: Comparative Analysis of Gene Expression Level by Quantitative Real-Time PCR Has Limited Application in Objects with Different Morphology
Source: PLoS One. 2012 May 30;7(5):e38161. doi: 10.1371/journal.pone.0038161 (PMC3364230; doi:10.1371/journal.pone.0038161)
Supplement: Table S1 — List of analysed genes, primers and different parameters derived from qRT-PCR analysis. (DOC) [file pone.0038161.s002.doc]

## Table S1. List of analyzed genes, primers and different parameters derived from qRT-PCR analysis.

| Name | Primer sequences | Tm (^o^C) | Amplicon length (bp) | Amplification efficiency |
| --- | --- | --- | --- | --- |
| AT4G34270 | GAAACGGTTGAGGCAAAAGCTAA | 62 | 153 | 1,97 |
|  | CATTGTCAGCCAGTTCATCTTCATA |  |  |  |
| AT5G25760 | TGGACCGCTCTTATCAAGGACC |  | 222 | 1,98 |
|  | AGCTCTACACACAGACTGAAGCGT |  |  |  |
| AP2 | AACATTGGTAGCGGAGGCGGATTCT |  | 199 | 1,98 |
|  | AGAGGAGGTTGGAAGCCATTTGTCTG |  |  |  |
| AP3 | TGGATACCAAATCGAAGGGTCACG |  | 119 | 1,96 |
|  | AGATGGAAGGTAATGATGTCAGAGGCA |  |  |  |
| PI | TTGAACATGGCCTCGACAAAGTCC |  | 206 | 1,96 |
|  | TTGGCTGAATCGGTTGCACTCT |  |  |  |
| AG | CTTATGCCACCACCTCAAACGCAA |  | 132 | 1,95 |
|  | ACACTAACTGGAGAGCGGTTTGGT |  |  |  |
| CLV1 | TCGGATGCTGCTATTGTTGTTGCG |  | 175 | 2,02 |
|  | TTCGCCACGGATTTAGGAGGGTTA |  |  |  |
| CLV2 | TGAGGATCACAGAGTTGAAGAGT |  | 395 | 2,01 |
|  | AAAGATACTTGAGGTTGGAACCAT |  |  |  |
| WUS | AGAAGAAGAATGTGGTGGCGATGC |  | 156 | 2,00 |
|  | AGACGTAGCTCAAGAGAAGCGCAA |  |  |  |
